# Impact of lymph node dissection on the prognosis of intrahepatic cholangiocarcinoma: a systematic review and meta-analysis

**DOI:** 10.3389/fonc.2025.1590019

**Published:** 2025-07-30

**Authors:** Jun Yu, Fan Zhou, Xing-Guo Tan, Jun Guo, Wei Feng

**Affiliations:** Department of Hepatobiliary Surgery, Yueyang Affiliated Hospital of Hunan Normal University, Yueyang, China

**Keywords:** intrahepatic cholangiocarcinoma, lymph node dissection, lymphadenectomy, prognosis, ICC

## Abstract

**Background:**

Intrahepatic cholangiocarcinoma (iCCA) is a malignant tumor second only to hepatocellular carcinoma in terms of incidence among primary liver cancers. Surgical resection is currently the preferred treatment for iCCA. However, the prognostic significance, complications, and clinical benefits of lymph node dissection (LND) in iCCA patients remain a topic of debate within the academic community.

**Methods:**

To evaluate the impact of LND on overall survival (OS) and prognosis in patients with resectable iCCA, studies published from various databases, including PubMed, Embase, Web of Science, and the Cochrane Library. A meta-analysis was conducted in accordance with the Preferred Reporting Items for Systematic Reviews and Meta-Analyses guidelines. The impact of LND on prognosis was analyzed.

**Results:**

A total of 5,787 patients from twenty-one retrospective cohorts were included in the final analysis. The results indicated that clinically node-negative patients who underwent LND had significantly better survival outcomes compared to those who did not undergo LND (*P*<0.01). In the R0 resection subgroup, LND was associated with improved survival compared to non-LND (*P*<0.01), while in the non-R0 resection subgroup, the LND group exhibited significantly fewer survival benefits than the non-LND group (*P*<0.01). When Compared to patients in the non-LND group, those in the LND N- group demonstrated significantly greater survival (*P*<0.05), while patients in the LND N+ group experienced significantly shorter OS (*P*<0.01).

**Conclusion:**

Patients with resectable iCCA who underwent LND had better survival outcomes compared to those who did not undergo LND. Therefore, routine LND should be performed for clinically lymph node-negative (cLNM-) iCCA patients.

**Systematic review registration:**

https://www.crd.york.ac.uk/PROSPERO/, identifier CRD42024564741.

## 1 Introduction

Intrahepatic cholangiocarcinoma (iCCA) is the second most common primary liver cancer after hepatocellular carcinoma and accounts for approximately 20% of all malignant liver tumors (1). Over the past two decades, the global incidence of iCCA has gradually increased, which may reflect both an increase in disease prevalence and an increasing trend toward early diagnosis (2). Surgical resection is considered the only treatment option that may extend patient survival, with approximately 20%-30% of patients being suitable candidates for this procedure (3). Lymph node metastasis (LNM) has been identified as a significant prognostic factor that affects iCCA outcomes (4), and lymph node dissection (LND) is crucial for managing this aspect of the disease. Despite controversial findings regarding its efficacy, routine LND for iCCA is recommended by the National Comprehensive Cancer Network (NCCN) guidelines (5, 6). Among the studies supporting LND, Umeda (4) et al. suggested that LND may significantly improve tumor prognosis, particularly in patients with hilar iCCA. However, LND does not appear to have a significant impact on survival rates among peripheral iCCAs compared with hilar carcinomas due to the greater potential of iCCA for intrahepatic or extrahepatic metastasis. Additionally, the study by Ke (7) et al. indicated that LND benefits clinical practice by guiding postoperative management specifically for iCCA patients with negative LNM results. Furthermore, research by Sposito (8) et al. demonstrated that adequate LND leads to improved survival outcomes among cN0 (clinically negative) iCCA patients with positive pathological lymph node negativity, thereby supporting the routine use of adequate LND in the treatment of patients with cN0 iCCA. In contrast, studies opposing LND, such as those by Kim (9) and Chang (10) et al. have shown that routine LND does not improve survival of iCCA patients. However, lymph node sampling remains valuable for accurate staging and plays a pivotal role in predicting outcomes and determining whether adjuvant therapy should be administered.

Due to some controversy remains regarding the beneficial effect and role of LND in post-iCCA patients, we performed this meta-analysis of published literature to assess the impact of LND on overall survival (OS) and prognosis among patients with resectable iCCA.

## 2 Materials and methods

### 2.1 Eligibility criteria

This study was reported in accordance with the 2020 Preferred Reporting Items for Systematic Reviews and Meta-Analyses and Assessing the Methodological Quality of Systematic Reviews guidelines (11, 12). The inclusion criteria were as follows: (1) Study type: Cohort studies including retrospective or prospective designs were considered. Publications from January 1, 2013, to September 31, 2024, were included regardless of country of origin or language (Chinese or English). (2) Subjects: All cases included in the literature had a confirmed diagnosis of iCCA through pathological analysis. (3) Interventions and Terminology Definitions: Patients were categorized into two groups: those who underwent LND and those who did not undergo lymph node dissection (nLND). Patients with LND are categorized based on pathological results into two groups: LND N+ (those undergoing LND with pathological confirmation of positive lymph nodes) and LND N- (those undergoing LND with pathological confirmation of negative lymph nodes). The classification of lymph node status is as follows: cLNM- indicates preoperative clinical lymph node negative status (cLNM- patients are defined as individuals who show no positive evidence of lymph node involvement through imaging examinations, including computed tomography (CT), ultrasound, positron emission tomography-computed tomography (PET-CT), and magnetic resonance imaging (MRI), as well as biomarkers such as CA 19-9); cLNM+ indicates preoperative clinical lymph node positive status; pLNM- indicates pathological lymph node negative status; and pLNM+ indicates pathological lymph node positive status. Finally, based on intraoperative surgical margins, the classifications are as follows: R0 indicates microscopically negative resection margins (complete tumor resection), and R1 indicates incomplete resection. (4) Outcome measures: OS (OS was defined as the time from randomization to death from any cause), DFS (DFS was defined as the time from randomization to disease recurrence or death from any cause), Kaplan–Meier survival curve, postoperative complications, and postoperative recurrence rate. The exclusion criteria were as follows: (1) duplicate publications; (2) sample size <60 patients; (3) nonprimary iCCA combined with other primary malignant tumors; (4) lack of relevant outcome indicators. And (5) The grouping method was inconsistent, as it was not clearly delineated into the LND group and the non-LND group.

### 2.2 Information sources and search strategy

By searching computer databases such as PubMed, Embase, Web of Science, the Cochrane Library, Wanfang, China Knowledge Network, and other relevant literature published in the past 10 years, the most recent literature was included, with a search time limit from January 1, 2013, to September 31, 2024. The search strategy was developed according to the Cochrane Collaboration’s handbook: the search terms included “Bile Duct Neoplasms,” “Cholangiocarcinoma,” “Intrahepatic cholangiocarcinoma,” “Biliary tract cancers,” “Intrahepatic bile duct carcinoma,” “iCCA,” “Lymph Node Excision,” “Lymphatic clearance,” and “Lymphadenectomy.”

### 2.3 Data collection process and data items

The Preferred Reporting Items for Systematic Reviews and Meta-Analyses standard was used to screen the literature. Two researchers independently read the literature, excluding obviously unqualified studies, such as duplicate literature, and then independently read the titles and abstracts of the literature obtained. They ensured that the literature met the inclusion criteria, and finally, they read the full texts carefully to determine final inclusion of each study. If any discrepancies were found during the screening process and if it was difficult to determine whether or not to include a study, its inclusion was discussed, sometimes by consultation with a third researcher. The extracted data included authors’ names, publication years, countries, sample sizes, patient sex, LND and nLND sample sizes, study types, matching methods, median follow-up times, multicenter study status, surgical margin status, OS, DFS, postoperative complications, and postoperative recurrence rate. Postoperative complications were evaluated according to the Clavien-Dindo classification of surgical complications (13).

### 2.4 Quality assessment of the literature

The Newcastle-Ottawa Scale was used to assess the quality of the literature. The quality assessment criteria included the selection of study subjects, comparability between groups, and measurement of exposure factors. Each study was scored from 0 to 9 points, and two researchers independently assessed the scores. Discrepancies were resolved through discussion and consultation with a third researcher. Cohort studies with scores ≥6 were considered to be of high quality, and studies with scores ≥4 were included in this meta-analysis.

### 2.5 Statistical analysis

RevMan 5.3 software was used for the data analysis. Patients were divided into the LND and nLND groups based on whether LND was performed. The LND group was further divided into a lymph node-positive group (N1 group) and a lymph node-negative group (N0 group). OS and DFS were used as effect sizes and are represented by hazard ratio (HR). For the included studies, the HR values for the risk rate were obtained through two approaches: 1. The risk rate values were explicitly given in the included literature. 2. If the risk rate values were not given in the original literature, Engauge-Digitizer data extraction software was used. The HR obtained from the preliminary study underwent multivariable adjustment. This software is used to extract data from target curves in the literature, and in this study, the target curve was the Kaplan–Meier survival curve. After extracting the target data, the risk rate was calculated using the risk rate calculation method summarized by Tierney, J.F (14). The postoperative recurrence rate and postoperative complication rate were analyzed using odds ratios. The chi-square test and I^2^ test were used to assess heterogeneity between study results. A *P*>0.1 and I^2^<50% indicated low heterogeneity between studies, and a fixed-effects model was used to combine effect sizes. A *P*<0.1 or I^2^>50% indicated heterogeneity between studies, and a random-effects model was used to combine effect sizes. Subgroup analysis was performed if necessary to explore the source of heterogeneity and to evaluate the reliability of the results. To control the family-wise error rate in subgroup analyses, *P*-values were adjusted using the Holm-Bonferroni method. All reported *P*-values in subgroup comparisons reflect this adjustment unless otherwise specified. A funnel plot was drawn to test for publication bias.

## 3 Results

### 3.1 Study selection and study characteristics

**Figure 1:**
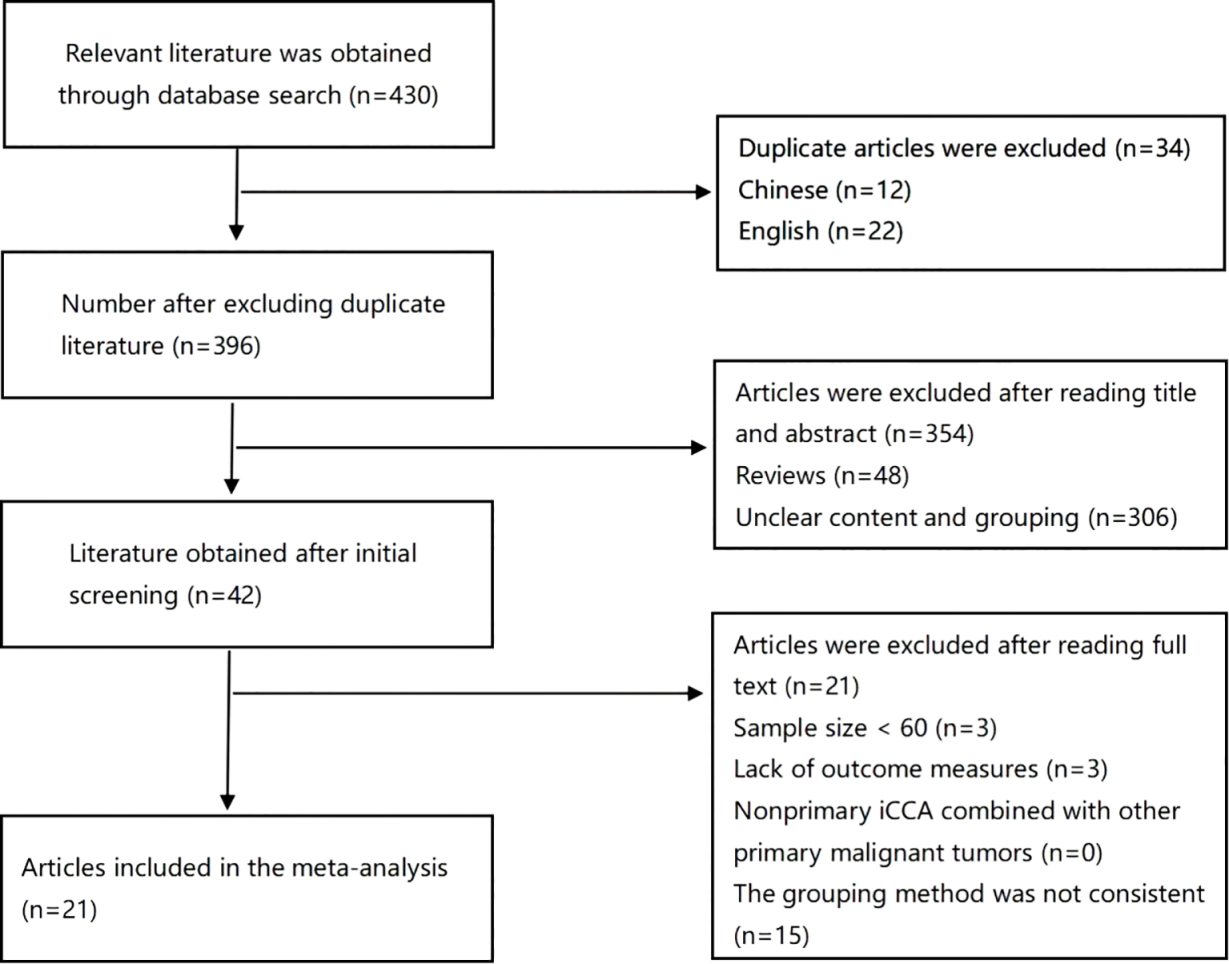
Diagram of the literature search and study selection.

The search results are summarized in **Figure 1**. In all, 430 articles were retrieved through database searches, 34 duplicate articles were excluded, 354 articles were excluded after reading the abstracts, and 21 articles were excluded after reading the full texts; finally, 21 eligible studies were included in the analysis (4, 5, 7, 9, 10, 15–30) with a total of 5787 patients. No discrepancies occurred between the researchers regarding inclusion and exclusion criteria. The characteristics of the 21 included studies are shown in **Table 1**. All included studies were retrospective, with 18 studies from Asia, including 13 from China (5, 7, 10, 15, 17, 19, 23, 24, 26–30), 2 from Japan (4, 20), 3 from South Korea (9, 18, 21), 2 from the United States (16, 22), and one joint study from Japan and France (25).

**Table 1:** Basic characteristics and quality scores of the included studies.

Study	Year	Country	Sample number	Group		Multicenter	Median follow-up (Months)	Type	Quality
				LND	nLND				
Chang ME (10)	2017	China	103	36	67	No	–	Retrospec	5
Chen C (15)	2023	China	442	221	221	Yes	–	Retrospec	8
Hu H(1) (5)	2021	China	232	177	55	Yes	19	Retrospec	8
Hu H(2) (16)	2021	America	2037	859	859	Yes	23	Retrospec	8
Hu J (17)	2017	China	422	73	349	No	–	Retrospec	6
Ke Q (7)	2021	China	212	106	106	Yes	–	Retrospec	7
Kim DH (9)	2015	Korea	215	113	102	No	17	Retrospec	6
Kim SH (18)	2019	Korea	68	34	34	No	65	Retrospec	7
Li DY (19)	2013	China	114	53	61	No	–	Retrospec	6
Miyata T (20)	2017	Japan	64	23	41	No	37	Retrospec	6
Navarro JG (21)	2020	Korea	210	160	50	No	24	Retrospec	8
Umeda Y (4)	2022	Japan	310	224	86	Yes	25.6	Retrospec	8
Vitale A (22)	2016	America	402	201	201	Yes	56	Retrospec	8
Wu ZF (23)	2015	China	85	50	35	No	–	Retrospec	5
Yang F (24)	2022	China	147	80	67	Yes	87	Retrospec	6
Yoh T (25)	2019	France Japan	112	56	56	Yes	28.4	Retrospec	8
Dai GF (28)	2021	China	109	58	51	No	35.1	Retrospec	6
Huang XJ (30)	2020	China	142	86	56	No	41	Retrospec	5
Ji HX (26)	2020	China	154	77	77	No	33.5	Retrospec	7
Tan ZG (29)	2021	China	135	72	63	No	32	Retrospec	6
Zhang HY (27)	2022	China	72	36	36	No	–	Retrospec	7

LND, lymph node dissection.

### 3.2 Survival outcomes and meta-analysis results

#### 3.2.1 Meta-analysis of LND and nLND patients

**Figure 2:**
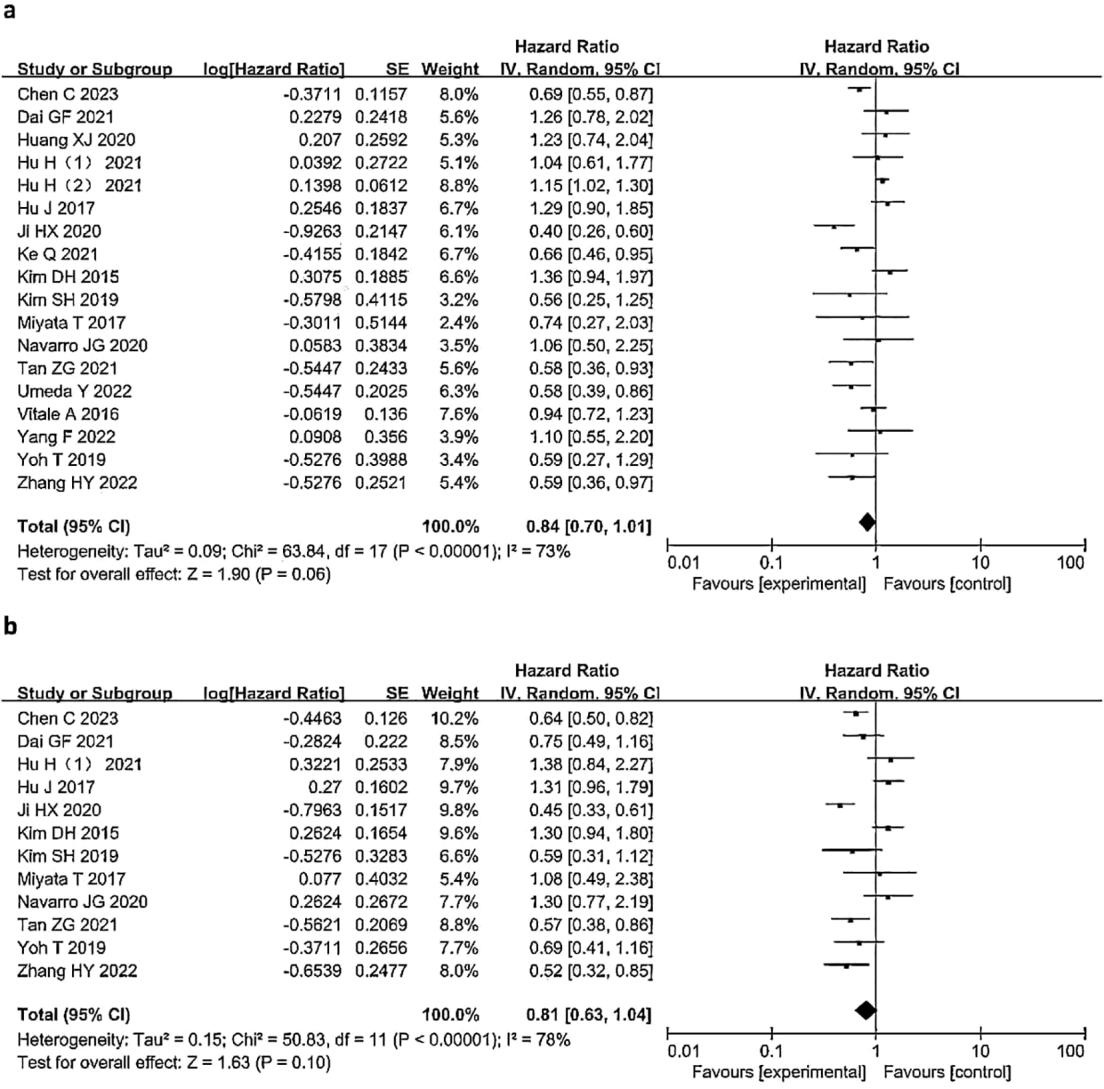
Forest plot comparing OS **(a)** and DFS **(b)** in the LND and nLNDgroups. LND, lymph node dissection; OS, Overall Survival; DFS, Disease-Free Survival; IV, Inverse variance method; CI, confidence interval; SE, standard error.

Eighteen studies (4, 5, 7, 9, 15–18, 20–22, 24–30) (18 studies,91.11%,5273/5787)were included in the analysis of OS (**Figure 2a**). Three studies (10, 19, 23) did not conduct a comprehensive survival analysis comparing LND and nLND, so they were excluded.High heterogeneity was observed between the studies (I^2^ = 73%, *P*<0.1), and a random-effects model was used for the analysis. The results indicated no significant difference in OS between the LND and nLND groups (HR=0.84, 95% CI: 0.70-1.01; *P*=0.06, *P*_adj_=0.0166).Twelve studies (5, 9, 15, 17, 18, 20, 21, 25–29) (12 studies,38.62%,2235/5787) were included in the analysis of DFS (**Figure 2b**). High heterogeneity was observed between the studies (I^2^ = 78%, *P*<0.1), and a random-effects model was used for the analysis. The results indicated no significant difference in DFS between the LND and nLND groups (HR=0.81, 95% CI: 0.63-1.04; *P*=0.10, *P*_adj_=0.025). The subgroup analyses for patients with cLNM- and R0 resection were pre-specified in our protocol based on clinical relevance. Subgroup analyses were conducted as follows.

**Figure 3:**
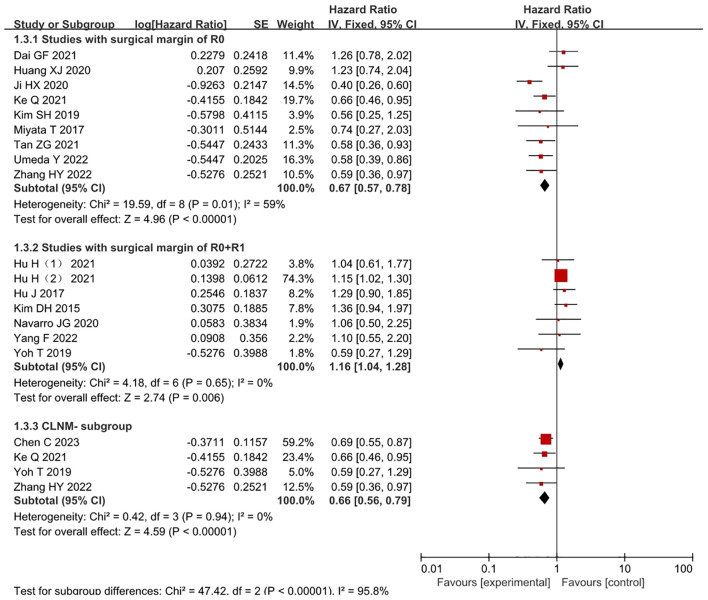
Forest plot comparing the OS in studies where the surgical margins were R0 or R0+R1 within the LND and nLND groups. And comparing OS in cLNM- patients. R0: negative surgical margin. R1: positive surgical margin. cLNM-, clinical lymph node metastasis-negative.

Among them, 7 studies (7, 15, 17, 25, 27, 28, 30) included only cLNM- patients (**Figure 3**). High heterogeneity was observed between the studies, and analysis revealed that 4 studies (7, 15, 25, 27) used matching methods (4 studies,14.48%,838/5787), such as propensity score matching, while the remaining 3 studies (17, 28, 30) did not use any matching methods. After excluding these studies, the heterogeneity significantly decreased (I^2^ = 0%, *P*=0.94), and a fixed-effects model was used for the analysis. The results indicated that the LND group had a survival benefit compared with the nLND group for patients in the cLNM- subgroup, and the difference was statistically significant (HR=0.66, 95% CI: 0.56-0.79, *P*<0.001, *P*_adj_=0.005).

Complete R0 surgical resection was performed in 9 studies (4, 7, 18, 20, 26–30) (**Figure 3**) (9 studies,21.88%,1266/5787). The results indicated that the LND group had significantly longer OS than the R0 resection subgroup within the nLND group (HR=0.68, 95% CI: 0.52-0.88; *P*<0.001, *P*_adj_=0.0056). R0+R1 surgical resection was performed in 9 studies (5, 9, 15–17, 21, 22, 24, 25). Compared with the same subgroup, the study by Chen C (15) included only lymph node-negative samples, while the study by Vitale A (22) did not provide detailed descriptions of surgical margins. After excluding these two studies (7 studies,58.32%,3375/5787), the heterogeneity decreased (I^2^ = 0%, *P*<0.01), and a fixed-effects model was used for the analysis. The results indicated that the LND group had significantly shorter OS than the non-R0 resection subgroup within the nLND group (HR=1.16, 95% CI: 1.04-1.28; *P*=0.006, *P*_adj_=0.0125).

#### 3.2.2 Meta-analysis of LND N- and nLND patients

**Figure 4:**
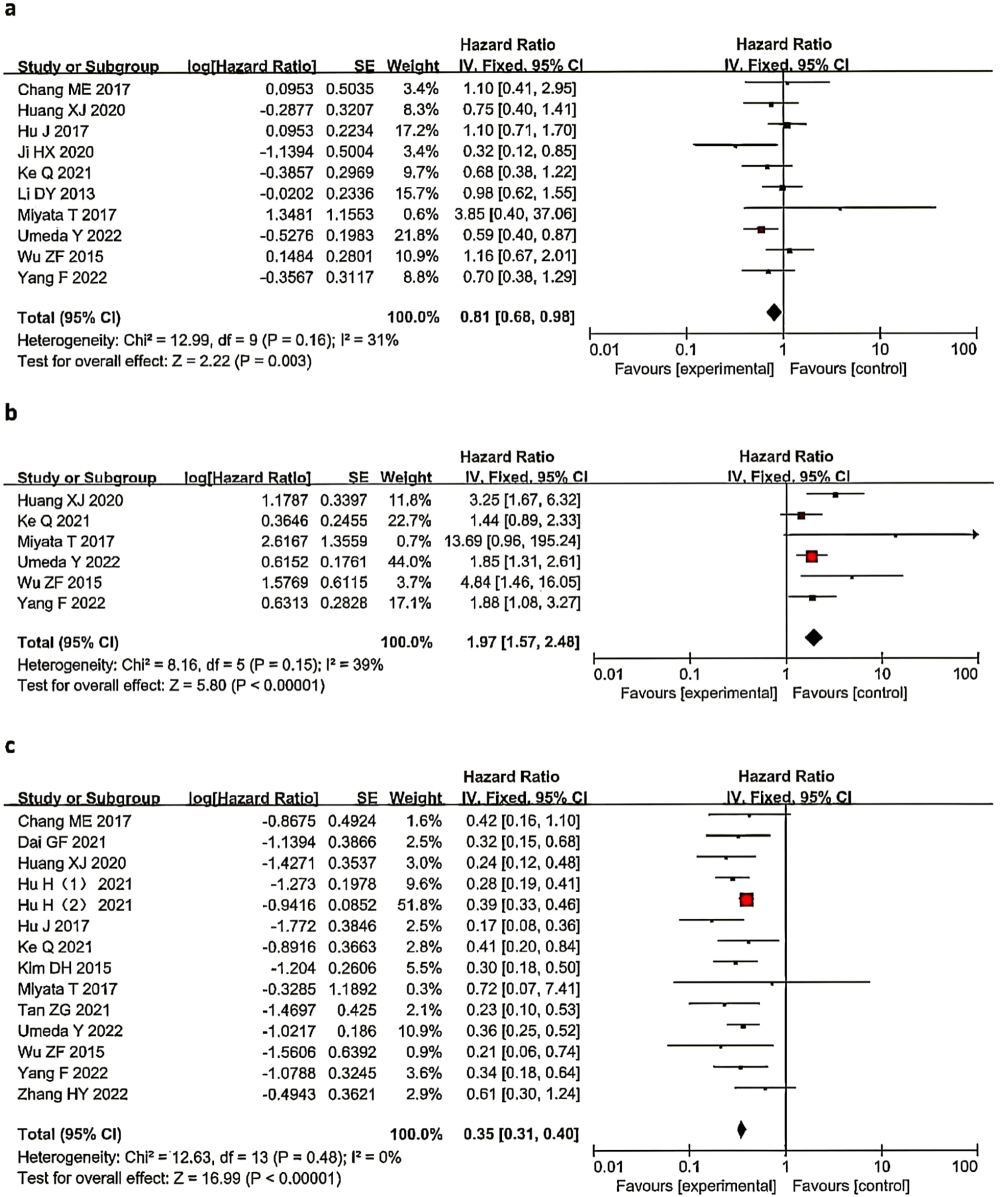
Forest plot comparing OS in the LND N- and nLNDgroups **(a)**. Comparing OS in the LND N+ and nLNDgroups **(b)** and comparing OS in the LND N- and LND N+ groups **(c)**. LND, lymph node dissection; N-, Negative lymph nodes; N+, Positive lymph nodes.

Ten studies (4, 7, 10, 17, 19, 20, 23, 24, 26, 30) (10 studies,29.98%,1735/5787) compared the OS of LND N- and nLND patients (**Figure 4a**). Low heterogeneity was observed between the studies (I^2^ = 31%, *P*=0.16), and a fixed-effects model was used for the analysis. The results indicated that the LND N- group had a greater survival benefit than the nLND group, and the difference was statistically significant (HR=0.81, 95% CI: 0.68-0.98, *P*=0.003, *P*_adj_=0.01).

#### 3.2.3 Meta-analysis of LND N+ and nLND patients

Seven studies (4, 7, 10, 20, 23, 24, 30) compared the OS of LND N+ and nLND patients (**Figure 4b**), and the interstudy heterogeneity was high (I^2^ = 54%, *P* < 0.1). The analysis revealed that the LND rate in the study by Chang (10) et al. (34.9%) was lower than that in other studies in the same group,heterogeneity decreased after exclusion(I^2^ = 39%, *P* < 0.01).The results of the fixed effects model (6 studies,16.58%,960/5787)showed that the OS of patients in the LND N+ group was lower than that in the nLND group, and the difference was statistically significant (HR=1.97, 95% CI: 1.57-2.48, *P* < 0.001, *P*_adj_=0.0063).

#### 3.2.4 Meta-analysis of LND N+ and LND N- patients

Fourteen studies (4, 5, 7, 9, 10, 16, 17, 20, 23, 24, 27–30) (14 studies,74.05%,4285/5787) compared the OS of LND N- and LND N+ patients (**Figure 4c**). Low heterogeneity was observed between studies (I^2^ = 0%, *P*=0.48), and a fixed-effects model was used for analysis. The results suggested that patients in the LND N- group had significantly longer OS than those in the LND N+ group (HR=0.35, 95% CI: 0.31-0.40; *P*<0.001, *P*_adj_=0.0071).

#### 3.2.5 Meta-analysis of postoperative complications and recurrence rate

**Figure 5:**
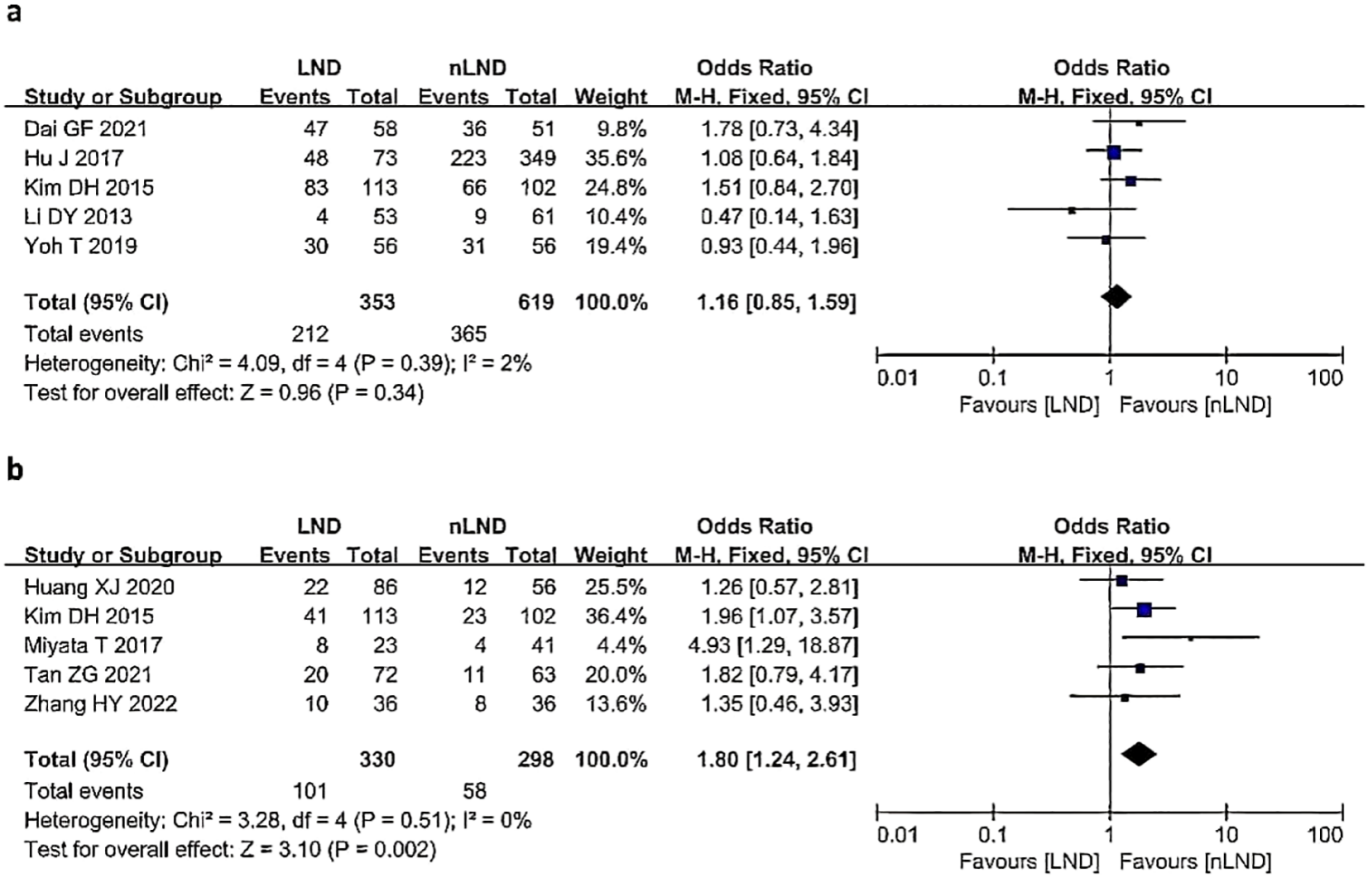
Postoperative outcomes following LND versus nLND in terms of recurrence **(a)** and postoperative morbidity **(b)**. LND, lymph node dissection; M−H, Mantel–Haenszel method; CI, confidence interval.

Five studies (9, 17, 19, 25, 28) (5 studies,16.79%,972/5787) reported the postoperative recurrence rate (**Figure 5a**), with low heterogeneity between studies (I^2^ = 2%, *P*=0.39). A fixed-effects model was used for analysis, and the results indicated no statistically significant difference in the postoperative recurrence rate between the LND group and the nLND group (HR=1.16, 95% CI=0.85-1.59, *P*=0.34, *P*_adj_=0.05).

Five studies (9, 20, 27, 29, 30)(5 studies,10.85%,628/5787) reported the incidence of postoperative complications (**Figure 5b**), with no heterogeneity among the studies (I^2^ = 0%, *P*=0.51). A fixed-effects model was used for analysis, and the results suggested that the LND group had a significantly greater incidence of postoperative complications than the nLND group (HR=1.80, 95% CI: 1.24-2.61;*P*=0.002,*P*_adj_=0.0083).

### 3.3 Publication bias

**Figure 6:**
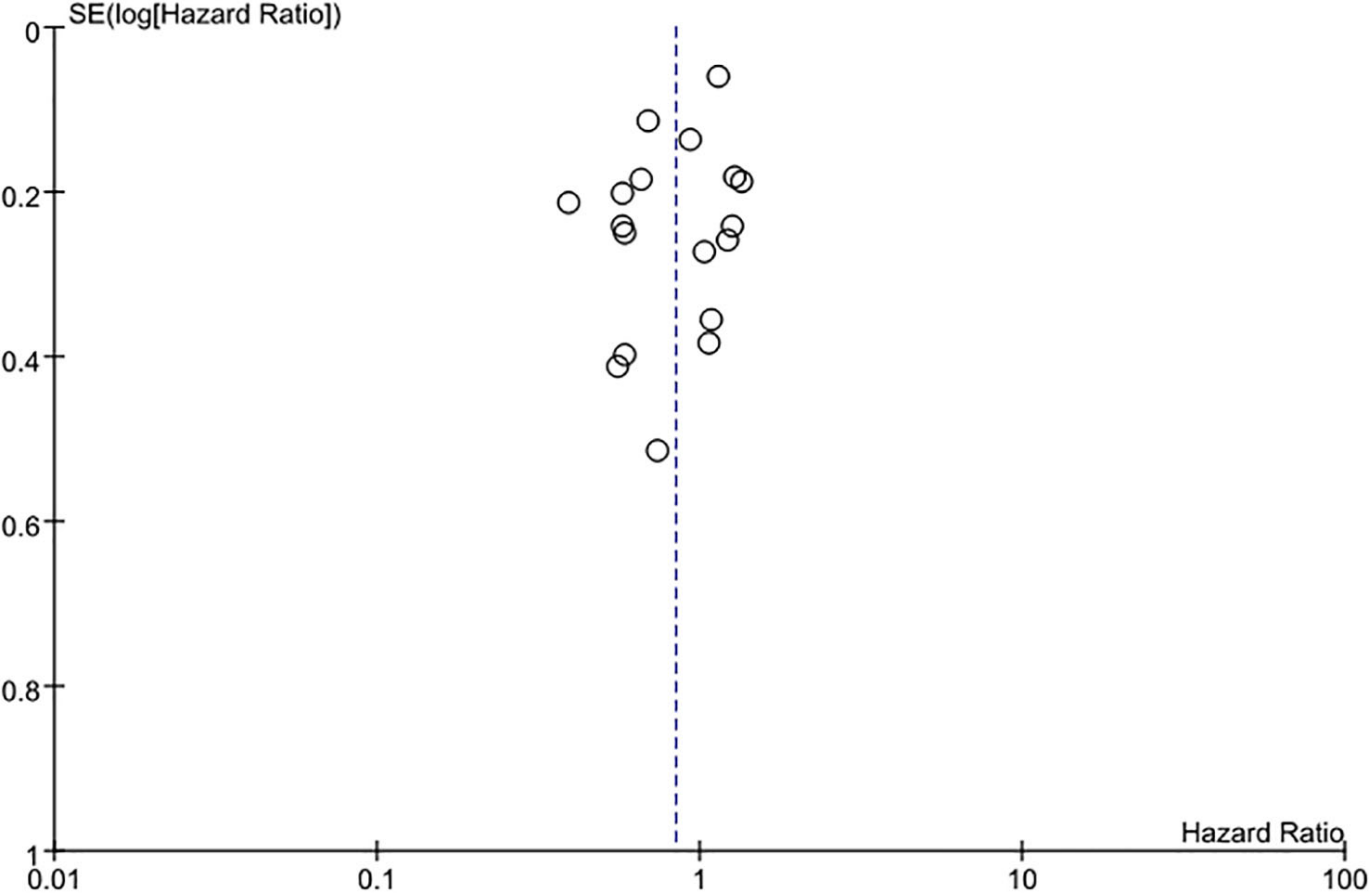
Funnel plot of OS in patients with intrahepatic cholangiocarcinoma meta-analysis.

The funnel plot of the studies included in this meta-analysis was relatively concentrated and symmetrical, which indicates no significant publication bias (**Figure 6**). To quantitatively assess publication bias, we performed Egger’s linear regression test for the primary outcome (OS). The results were as follows: OS analysis (Egger’s intercept =1.32, 95%CI: -0.87-3.51, *P*=0.184). These findings confirm no significant asymmetry in the funnel plots, aligning with our initial conclusion that publication bias is unlikely to substantially affect the results.

## 4 Discussion

iCCA is the second most common malignant primary liver cancer, as it accounts for approximately 20% of all malignant liver tumors (1). iCCA is relatively rare in clinical practice and has an overall 5-year survival rate of less than 10%. In recent years, the incidence and mortality rates of iCCA have continued to increase (31). Surgical resection is currently the preferred treatment for iCCA, but early diagnosis is difficult, and only 20-40% of patients have the opportunity for surgical resection. The postoperative recurrence rate is high, with a 5-year recurrence rate up to 80% and a 5-year survival rate less than 20% (32–34). LNM, with a rate of 45-60%, is one of the most important adverse prognostic factors for curative surgery in iCCA patients (31). LND has important value in the diagnosis of LNM, tumor staging, prognostic guidance, and adjuvant treatment for iCCA. The prognostic value, complications, and clinical benefits of LND in iCCA patients are currently a focus of debate in the academic community.

This study presents a quantitative analysis of the effectiveness and safety of LND in iCCA patients. In all, 21 articles and 5781 patients were included in the analysis. These studies included both single-center and multicenter studies, all of which were retrospective. Most of these studies described the range of LND but did not specify the number of lymph nodes dissected. These patients received curative R0 surgical treatment regardless of their cLNM- status, as assessed by imaging and discussion among clinical doctors.

The results of the analysis suggested that compared with patients who underwent nLND, patients who underwent LND did not experience a survival benefit. Due to its significant heterogeneity, subgroup analyses were conducted to investigate the sources of this variability (for example, R0 versus non-R0 resection, cLNM status, etc.). These analyses revealed that factors such as the status of surgical margins and the accuracy of preoperative lymph node staging contributed to the observed heterogeneity. Subgroup analysis suggested that patients diagnosed with cLNM- before surgery had better survival outcomes with LND than nLND. Hu J et al. (17) reported that routine LND may not improve the survival rate of patients with resectable iCCA and cLNM-. The analysis of various studies revealed that the cLNM- group also included some N+ patients. Chen C et al. (15) included all patients with pLNM- and found that patients in the LND group had better OS and DFS. The sensitivity, specificity, and accuracy of PET/CT vs. MRI for the diagnosis of regional LNM were 70.0% vs. 50%, 91.7% vs. 83.3%, and 81.8% vs. 68.2%, respectively (35). With improvements in imaging technology and the increase in the specificity of experimental indicators, accompanied by a decrease in the false-negative rate of LNM, cLNM- patients may benefit from LND. In addition, subgrouping based on surgical margin status, R0 and R0+R1, suggested that the survival benefit of LND was significantly greater than that of nLND in the R0 group but was smaller in the R0+R1 group than in the nLND group. This suggests that the surgical margin status affects the prognosis of patients undergoing LND. Consequently, for patients with iCCA who have undergone non-R0 resection, it is our position that lymphadenectomy may be deemed unnecessary, given that these tumors are predominantly at an advanced stage and exhibit vascular invasion.

The analysis of 10 studies revealed that patients with pLNM- had better survival outcomes if they underwent LND(i.e., LND N-) than those who underwent nLND, and the analysis of 14 studies showed that patients with pLNM- who underwent LND also had significantly better survival prognosis than those with pLNM+. These data further suggests that routine LND should be considered for patients with cLNM-negative iCCA. This study also revealed that the OS of patients with LNM who underwent LND was shorter than that of patients with lymph node negativity and that of patients who underwent nLND. Ke et al. (7) reported that patients who underwent LND were more likely to receive postoperative adjuvant treatment, and compared with pLNM- and nLND patients, pLNM+ patients had a worse prognosis (*P*<0.05), but only LNM+ patients benefited from postoperative adjuvant treatment (*P*<0.05). This indicates that LND may also play an important role in the postoperative management of iCCA patients. For such high-risk patients, postoperative adjuvant radiotherapy may further decrease the likelihood of local recurrence. For instance, the Mayo regimen, which combines external radiation with brachytherapy, has demonstrated an increase in the 5-year survival rate for hilar cholangiocarcinoma, indicating its potential applicability to iCCA (36). In addition, Chafoori et al. (37) assert that for advanced and unresectable tumors, radiotherapy and chemotherapy can achieve local control of extrahepatic cholangiocarcinoma for up to two years. Similarly, Esmail et al. (38) proposed that integrating various treatment modalities, such as combining immunotherapy with chemotherapy, may result in improved survival outcomes. Statistical analysis indicates that patients diagnosed with pLNM+ who undergo lymphadenectomy experience less benefit from the procedure compared to those who do not. It is important to note, however, that the majority of patients in the pLNM+ cohort often present with compromised physical conditions, advanced tumor stages characterized by vascular and serosal invasion, and a higher incidence of complications. Despite these challenges, it has been observed that patients with pLNM+ can achieve improved outcomes through adjuvant therapy. Consequently, it is imperative to engage in a discussion regarding the appropriateness of lymphadenectomy for patients with positive LNM. A thorough preoperative assessment should be conducted, taking into account variables such as age, nutritional status, tumor size and quantity, the extent of LNM, CA19-9 levels, and any existing comorbidities. If the advantages of postoperative adjuvant therapy surpass the potential detriments associated with lymphadenectomy, the procedure should be considered; conversely, if the disadvantages of lymphadenectomy outweigh the benefits of postoperative adjuvant therapy, the procedure should be avoided. Achieving a balance between these two considerations necessitates further experimental research, which will be a focus of our future investigations.

This study revealed no significant difference in the postoperative recurrence rate between LND and nLND patients (I^2^ = 2%, *P*=0.34, *P*_adj_=0.05). However, the analysis revealed that LND significantly increased the incidence of postoperative complications compared with nLND. According to various studies, patients who undergo LND may have longer surgery times, greater blood loss, and longer drainage tube removal times. The occurrence of postoperative complications is one factor that contributes to a poor prognosis in cancer treatment (39). Therefore, iCCA patients should pay attention to the indications for LND, ensure safe surgery, and reduce postoperative complications to improve patient prognosis.This inquiry highlights a critical clinical consideration regarding the equilibrium between the advantages of survival and the potential risks associated with postoperative complications. Among the five studies examined in relation to postoperative complications, three (27, 29, 30) addressed the prognostic significance of LND in patients with cLNM-. The findings suggested that although LND may prolong the duration of surgery and the length of postoperative hospitalization, there was no notable difference in intraoperative blood loss or the incidence of postoperative complications. Moreover, for patients with iCCA and cLNM-, prophylactic LND appears to offer greater benefits than risks and can be integrated as a standard surgical procedure. It is posited that the implementation of standardized protocols and the application of proficient surgical techniques are vital for the successful execution of the surgery. Additionally, the patient’s capacity to endure the surgical procedure is a fundamental prerequisite, and conducting a thorough preoperative assessment may mitigate the likelihood of postoperative complications in iCCA patients, thereby enhancing their prognosis.

Currently, the practices for LND during the treatment of iCCA are not standardized. This lack of standardization reflects a lack of guidelines, which can be attributed to conflicting evidence in the literature. Insufficient evaluation of lymph node status may lead to inaccurate staging and insufficient application of adjuvant chemotherapy. Zhang et al. (40) demonstrated that at least 6 LNs should be routinely dissected and that examination beyond the 12 stations should be performed to obtain the maximum opportunity for accurate staging. Based on their recommendation, they proposed a new N stage model: N_0_ (LNM 0), N_1_ (LNM 1-2), and N_2_ (LNM > 2). Similarly, Sposito et al. (8) also suggested that adequate LND, which involves the resection of at least 6 lymph nodes during liver resection, provides significant benefits for patients with less advanced tumors (i.e., isolated tumors, maximum tumor size <5 cm, CA 199 <200 U/ml, no evidence of major vessel invasion). In patients with these characteristics, the possibility of systemic disease dissemination is low, and the tumor may be localized to the liver and regional lymph nodes. Therefore, adequate LND can eliminate all micrometastatic disease, thereby improving recurrence-free survival and OS. Kim et al. (41) attempted to determine the minimum number of lymph nodes required for iCCA using a Bayesian Weibull model. Their results suggested that at least 5 LNs should be dissected in patients undergoing curative iCCA surgery to promote accurate staging. Chen et al. (15) reported that clearing more than 8 lymph nodes was associated with a better patient prognosis, and not only accurate staging, without increasing the incidence of postoperative complications or the length of hospital stay. The range of LND is still controversial, and further research studies may be needed to standardize the scope of LND. The liver has multiple lymphatic drainage pathways, which may also require further research, including the concept of sentinel lymph nodes.

This meta-analysis does have some limitations. First, all the studies included are retrospective, highlighting the scarcity of prospective research in this field. This limitation makes it challenging to control for external factors. Second, while frameworks such as the 8th edition AJCC staging system (42) and NCCN guidelines (6) recommend lymph node evaluation for iCCA, they do not provide granular specifications for the extent of LND (e.g., minimum lymph node yield or anatomical stations to dissect). Consequently, the indications and scope of LND remain surgeon-dependent, guided by institutional protocols, clinical experience, and preoperative imaging. This variability complicates cross-study comparisons of LND outcomes. Moreover, factors such as the experience of surgeons, institutional protocols for adjuvant therapy, and the heterogeneity in patient selection may inevitably confound the observed survival benefits.Third, most studies have focused on the prognosis of iCCA patients after LND and have neglected the role of surgical margins, tumor size and location, CA19-9, CEA, number of LNM, and number of lymph nodes dissected in the prognosis of iCCA patients with LND.

Our research findings advocate for routine LND in patients with cLNM - iCCA who undergo R0 resection, while cautioning against the use of LND in non-R0 cases. This stratified approach optimizes survival benefits while minimizing unnecessary morbidity. Compared to previous meta-analyses, our study innovatively addresses a long-standing controversy by: (1) integrating large-scale data; (2) isolating the cLNM cohort to quantify the true benefits of LND; (3) introducing resection margin status as a key stratification variable; and (4) employing p-value adjusted subgroup analyses to enhance reliability. These advancements provide a roadmap for updating clinical guidelines and standardizing surgical practices.

## 5 Conclusion

In summary, current evidence suggests that iCCA patients who undergo curative surgery benefit more from LND than from nLND, a procedure that is accompanied by improvements in imaging technology and experimental indicator specificity. cLNM- patients will also benefit from LND, and routine LND should be performed on cLNM- iCCA patients. This study revealed that more postoperative complications are associated with LND than with nLND; however, with limited sample data, more prospective studies are needed to standardize the indications and scope of LND, reduce postoperative complications, and improve prognosis.
